# Ecological-niche modeling reveals current opportunities for *Agave* dryland farming in Sonora, Mexico and Arizona, USA

**DOI:** 10.1371/journal.pone.0279877

**Published:** 2023-01-20

**Authors:** Hector G. Ortiz Cano, Robert Hadfield, Teresa Gomez, Kevin Hultine, Ricardo Mata Gonzalez, Steven L. Petersen, Neil C. Hansen, Michael T. Searcy, Jason Stetler, Teodoro Cervantes Mendívil, David Burchfield, Pilman Park, J. Ryan Stewart

**Affiliations:** 1 The Holden Arboretum, Kirtland, Ohio, United States of America; 2 Department of Research, Conservation and Collections, Desert Botanical Garden, Phoenix, Arizona, United States of America; 3 Department of Animal and Rangeland Sciences, Oregon State University, Corvallis, Oregon, United States of America; 4 Department of Anthropology, Brigham Young University, Provo, Utah, United States of America; 5 Instituto Nacional de Investigaciones Forestales, Agrícolas y Pecuarias (INIFAP), Campo Experimental Costa de Hermosillo, Sonora, México; 6 Floriculture Research Division, National Institute of Horticulture and Herbal Sciences, Rural Development Administration, Jeonju, South Korea; Soil and Water Resources Institute ELGO-DIMITRA, GREECE

## Abstract

For centuries, humans occupying arid regions of North America have maintained an intricate relationship with *Agave* (Agavoideae, Asparagaceae). Today *Agave* cultivation, primarily for beverage production, provides an economic engine for rural communities throughout Mexico. Among known dryland-farming methods, the use of rock piles and cattle-grazed areas stand out as promising approaches for *Agave* cultivation. Identifying new cultivation areas to apply these approaches in Arizona, USA and Sonora, Mexico warrants a geographic assessment of areas outside the known ranges of rock piles and grasslands. The objective of this study was to predict areas for dryland-farming of *Agave* and develop models to identify potential areas for *Agave* cultivation. We used maximum entropy (MaxEnt) ecological-niche-modeling algorithms to predict suitable areas for *Agave* dryland farming. The model was parameterized using occurrence records of Hohokam rock piles in Arizona and grassland fields cultivated with *Agave* in Sonora. Ten environmental-predictor variables were used in the model, downloaded from the WorldClim 2 climate database. The model identified potential locations for using rock piles as dryland-farming methods from south-central Arizona to northwestern Sonora. The *Agave*-grassland model indicated that regions from central to southern Sonora have the highest potential for cultivation of *Agave*, particularly for the species *Agave angustifolia*. Results suggest that there are many suitable areas where rock piles can be used to cultivate *Agave* in the Sonoran Desert, particularly in the border of southeastern Arizona and northwest Sonora. Likewise, cattle-grazing grasslands provide a viable environment for cultivating *Agave* in southern Sonora, where the expanding bacanora-beverage industry continues to grow and where different *Agave* products (e.g., syrups, fructans, saponins, and medicinal compounds) can potentially strengthen local economies.

## Introduction

The deep-rooted symbiotic relationship between the *Agave* genus and indigenous groups and rural communities in arid regions has been crucial to creating sustainable agroecosystems in arid regions in Mexico and North America [1, 2]. In the Sonoran Desert, beginning in pre-Columbian times, *Agave* was cultivated and used for centuries as an unabated source of food, drink, medicine, and fiber [2–5]. Desert farmers, such as the Hohokam, innovated dryland-farming techniques to cultivate *Agave* using rainfall runoff and rock mulching, also known as Hohokam rock piles [2, 4, 6–9]. Hohokam dryland farming made the extensive cultivation of *Agave* possible as a staple crop in the Sonoran Desert [2, 4]. Principles of *Agave* dryland farming, which enabled the Hohokam to successfully cultivate *Agave*, can be applied to the modern *Agave* agricultural industry, which is currently impacted by rising global temperatures and droughts [6, 10–12].

*Agave* cultivation in Hohokam rock piles ended before European contact [13]. However, several indigenous groups continued using *Agave* for food and beverage after the demise of the Hohokam civilization [5, 13]. After the Hohokam, the Opata and Pima tribes continued using *Agave* as a crop, including through the use of roasting pits to cook *Agave* to craft beverages in the Sierra Madre Occidental Mountains [5, 14–17]. In the early 1900s, rustic distilled drinks made from *Agave angustifolia* in the Sierra Madre Occidental in Sonora, Mexico were identified by the name of mescal bacanora [15, 17]. In the last 30 years, the bacanora industry and *A*. *angustifolia* as a crop have risen in economic prominence in the pueblos of the Sierra Madre Occidental, which can also be seen throughout the borderlands of Arizona, USA and Sonora [11, 17–19]. In 2019, the bacanora industry in Sonora produced 360,000 L of bacanora on 500 ha of land, with an estimated annual revenue of $3,392,640 [20]. Current trends of land use for cultivating *A*. *angustifolia* for bacanora in Sonora has been estimated to expand 100 ha annually [20]. The European Union now recognizes mescal bacanora as a protected regional-based product of the state of Sonora [21]. However, to continue cultivating *A*. *angustifolia* for bacanora and to promote *Agave* as crop in the borderlands of Sonora and Arizona, it is vital to find sustainable cultivation methods for *Agave* to thrive as the climate continues to change and to reduce the use of irrigation water in the rainfall-limited region [22].

Increasing temperatures and droughts in the American Southwest will continue to constrain yields of irrigated C_3_ and C_4_ crops [22–24]. However, crops that use crassulacean acid metabolism (CAM) as their primary photosynthetic pathway, such as those in the *Agave* genus, are best suited to be cultivated in semi-arid and arid climates [10, 25, 26]. *Agave* cultivation using dryland-farming techniques, first implemented in pre-Columbian times, has great potential in the Sonoran Desert region, which straddles the border between Sonora and Arizona [4, 22, 27, 28]. However, producers of modern-day bacanora do not appear to be using dryland-farming techniques, such as rock mulching, for cultivating *Agave* in the Sonoran Desert region [29–31]. In addition, commercial *Agave* plantations do not currently exist in Arizona or other parts of the U.S. [32].

*Agave* cultivation in the borderlands of Sonora with Arizona, is in the early phases of development [22, 33]. Northern-Sonoran *Agave* farmers are transitioning from a more traditional-rustic mescal industry to a more intensive and mechanized approach (i.e., use of tissue-culture facilities, greenhouses, and nurseries to propagate and produce plants; and use of modern distillation equipment for mescal production) to produce mescal [15, 22, 30, 33, 34]. Two dryland-farming systems to cultivate *Agave*, rock-piles and cattle-grazing areas, show promise to offset the impacts of current climate challenges in the U.S.-Mexico borderlands region in the Sonoran Desert. *Agave* rock piles were used extensively by the Hohokam in Arizona to cultivate *Agave murpheyi* and *Agave sanpedroensis* [4, 8, 27, 35]. Rock piles modified the soil microtopography, increasing rainfall-water catchment, infiltration, and soil microflora, which favored water and nutrient uptake of agaves [36–40]. In addition, rock piles acted as a barrier that protected *Agave* from natural predators [41]. In more modern times, *Agave* has been successfully cultivated in cattle-grazing areas (commonly called agostaderos in Spanish) in rural areas of Sonora, such as grassland pastures, since the early 1990s [15, 31]. Cattle-grazing areas with *A*. *angustifolia* are open areas with sandy-loam soils and medium-to-low stone content, in which shrubs, forbs, trees and native grasses are used for cattle foraging [15, 34]. In these areas, some species act as nurse plants, providing shelter and nutrients for *A*. *angustifolia* [34]. Using cattle-grazing areas enables Sonoran *Agave* farmers to diversify land use and income streams by raising livestock and *Agave* for producing bacanora. Incorporating *Agave* cultivation, which primary relies on rainfall instead of irrigation, would require only a minimum investment to implement [31, 34]. Nevertheless, despite the potential of these two dryland-farming systems, *Agave* cultivation in the Sonoran Desert is a notably under-utilized opportunity to expand the agricultural economies in the borderlands region of the U.S and Mexico [10, 42].

Given the high degree of water scarcity in the borderlands region, coupled with a lack of infrastructure to establish and maintain irrigation systems, a clear need exists to identify potential areas suitable for *Agave* dryland farming. Forecasting areas for *Agave* dryland farming could clearly benefit our understanding of *Agave* as a viable crop and help to expand its cultivation in the region. Making informed decisions on the use of land and conservation in the Sonoran Desert requires an assessment of suitable areas for potential *Agave* cultivation. This assessment is crucial given that predicted heat waves and droughts will increase in frequency and duration throughout the borderlands in the coming decades [43, 44].

Potential areas for dryland farming can be identified through remote-sensing techniques and ecological-modeling platforms, which use suitability-modeling techniques for predicting areas based on known dryland-farming locations and environmental aspects of the region (e.g., temperature, elevation, slope, aspect) [45]. Field observations can document the occurrence of plant or animal species, environmental attributes, or agricultural features that can be effectively used in geographic-distribution models. This kind of approach offers a low-cost, first-approach option to evaluate suitable areas for *Agave* dryland-farming. In order to identify suitable climate and areas that potentially can support *Agave* dryland-farming in the region, we used the Maximum Entropy (MaxEnt) modeling platform [46] to identify areas that are suitable for *Agave* dryland cultivation in southern Arizona and Sonora. MaxEnt uses ecological-niche theory [47] and maximum-entropy principles [48, 49] to create a probabilistic model of the occurrences and geographic extent of the species in a selected area using geographic locations and environmental information from a given area. We chose MaxEnt because it has been widely used in several research fields to create geographic-distribution models of animals, insects, wild plant species, crop species, and archaeological features [46, 48, 50–54]. In addition to modeling distributions of rock piles and cattle-grazed areas for potential *Agave* cultivation, MaxEnt also offers an accurate, simple, and information-rich analysis of environmental factors to forecast suitable climates to cultivate *Agave* using dryland farming [53, 54]. Forecasting potential suitable areas for *Agave* dryland farming is crucial for supporting local economies, which are reliant on agricultural production and distribution of *Agave* throughout the region [22, 33].

To identify new potential areas for *Agave* cultivation using dryland-farming techniques, we created suitability models using MaxEnt, which were based on existing Hohokam rock piles in Arizona and in cattle-grazed areas in Sonora where agaves were cultivated. We also used MaxEnt to evaluate if dryland-farming areas coincide with *Agave* natural habitats. We made models of 1) *Agave parryi*, a species endemic to northern Sonora and southern Arizona, which is not cultivated, but has potential to produce biofuel, phytochemicals, and mescal [55, 56], 2) *Agave palmeri*, which traditionally has been used to produce mescal in northern Sonora and studied for medicinal uses [55, 57, 58], and 3) *A*. *angustifolia*, which is extensively used to produce mescal within the protected designation-of-origin region for mescal bacanora in the Sierra Madre Occidental Mountains in Mexico. In developing these models, we had three main goals. First, assess the performance of MaxEnt modeling as a tool to predict suitable dryland-farming areas in Arizona and Sonora. Second, analyze individual models to identify potential areas based on their environmental suitability for *Agave* dryland farming. Third, compare models to determine which dryland-farming technique (i.e., rock piles or cattle-grazed cultivation) is most suitable for specific areas.

## Materials and methods

### The study area

**Fig 1:**
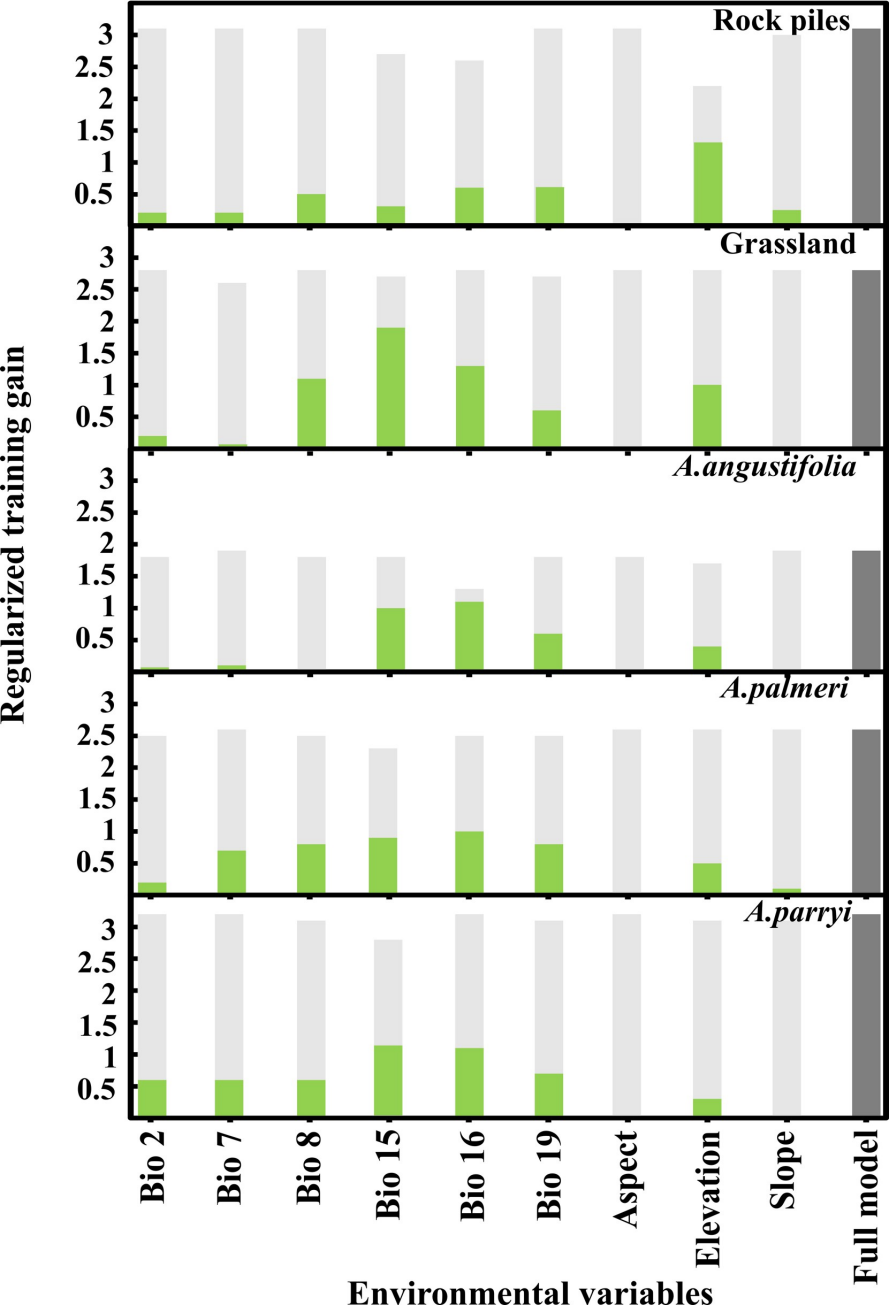
Summary of the importance of environmental variables in the development of suitability models for *Agave* dryland-farming (a, b) and *Agave* species (c, d, e) relative to regularized training gain. Green bars indicate model gain when only including individual environmental variables. Light-grey bars show the gain when the individual environmental variables are excluded from the full model. Dark-grey bars indicate the gain achieved in the full model, including all environmental variables in the model. Abbreviated environmental variables are defined as follows: Bio 2 = mean diurnal range (mean of monthly (maximum temperature—minimum temperature)), Bio 7 = temperature annual range (maximum temperature–minimum temperature), Bio 8 = mean temperature of wettest quarter, Bio 15 = precipitation seasonality, Bio 16 = precipitation of wettest quarter, and Bio 19 = precipitation of coldest quarter.

We selected an area encompassing Arizona and Sonora and adjacent states in the borderlands of the U.S. Southwest and northwestern Mexico (Fig 1). We included the states that share borders with Arizona, which comprise southeastern California, southern Utah, southern Nevada, and western New Mexico. In Mexico, we included eastern Chihuahua and northern Baja California.

### MaxEnt species distribution modeling

We used MaxEnt software version 3.4.4. [46] to create distribution models for where *Agave* may have been cultivated in rock piles and grasslands and where there might be potential cultivation of relevant crop plants within the *Agave* genus. Developing distribution models in MaxEnt involves inputting known geographic locations where samples have been found and relevant environmental variables (e.g., rainfall, temperature, elevation, aspect, etc.) to produce a probability map of habitat suitability [46, 54]. In the MaxEnt software, species, geographic location of localities, agricultural features, and plant or animal species can be processed with as little as three samples available in a known area [59].

### Dryland-farming occurrences: Rock piles and *Agave* grassland samples

We used geographic information from two dryland-farming systems for *Agave* in this study: 1) Hohokam rock piles in Arizona and 2) grassland fields cultivated with *A*. *angustifolia* in Sonora. A global-positioning-system (GPS) unit (Oregon 600, Garmin, Olathe, KS, USA) was used to record the location of rock piles at archaeological sites in Arizona using Universal Transversal Mercator (UTM) WGS84 as a coordinate system. Forty-four Hohokam agave rock piles were sampled at archaeological rock-pile fields in southern and central Arizona. The selected sites were previously reported in the literature [4, 9, 27, 60, 61] (Table 1). Field surveys were conducted between 2017 and 2019 with the assistance of Hohokam rock-pile experts, emeritus anthropologists Paul and Suzanne Fish, of the Arizona State Museum and Desert Laboratory on Tumamoc Hill.

**Table 1:** Sites and numbers of samples evaluated in our study of rock piles (top portion of table) and grasslands cultivated with *Agave angustifolia* (bottom portion of table) using MaxEnt.

**Archaeological sites with rock piles in Arizona, USA**	**Samples per site**
Tumamoc Hill, southern AZ	12
Marana, southern AZ	10
Santan Mountains, central AZ	4
Horseshoe Lake, central AZ	18
**Grasslands cultivated with *Agave* in Sonora, Mexico**	**Samples per site**
Mátape, central SON	1
San Pedro de La Cueva, central SON	1
Bacanora, central SON	1
Pueblo de Álamos, Ures, central SON	1
Álamos, southern SON	1
Tepache, northern SON	1
Villa Hidalgo, northern SON	2
Arizpe, northern SON	1
Banamachi, northern SON	1
Moctezuma, northern SON	5

We also identified geographic locations of grassland fields where *A*. *angustifolia* [3] is cultivated through an inventory of wild and cultivated *Agave* species conducted in Sonora [62, 63]. This is the most recent inventory made of wild *A*. *angustifolia* species after the one made by [3]. The inventory includes *A*. *angustifolia* commercial plantations for bacanora in the Sierra Madre Occidental Mountains in Sonora [15] (Table 1).

### *Agave* sample occurrences

We selected *Agave parryi* [3, 55] due to its long association with pre-Columbian *Agave* cultivation at archaeological sites in east-central Arizona and its potential use for mescal in the borderlands of Sonora and Arizona [33, 55, 56, 64, 65]. We also selected *Agave palmeri* [3, 55] because of its use in producing an artisanal mescal commonly called lechuguilla in northern Sonora [55, 57, 66]. Moreover, both species are endemic to the borderlands of Arizona and Sonora [1, 3, 33, 55, 57, 66]. We collected *A*. *parryi* and *A*. *palmeri* geographic location data from an online herbaria database [67]. In addition, geographic locations of wild *A*. *angustifolia* species were identified through an inventor of wild and cultivated *Agave* in Sonora [62, 63].

### Preprocessing of occurrences for MaxEnt modelling

Since the distance between rock-pile clusters at archaeological sites vary [2, 68], we used the ‘thin’ function of the spThin R statistical package to subsample rock piles from all the sites sampled [69]. The subsampling method, also called spatial thinning, via ‘thin’ function, uses an algorithm that analyzes the distance between sample occurrences and identifies those that reduce predictability of ecological niche models. The algorithm removes samples that overlap or highly correlate in a study area. The spatial thinning method enables users to retain the largest occurrence records while reducing bias sampling and autocorrelation among sample records [69]. We assessed autocorrelation between samples using the software RStudio Version 3.6.3 (R Development Core Team, 2020) (S4 Fig). The optimal-setting parameters for running MaxEnt were extracted using the ENMeval R package [70] for each dryland-farming system and wild *Agave* population selected for the study to ensure generation of models with high predictive power. MaxEnt model settings were extracted from predictor variables and sample occurrences using ENMTools in RStudio [71].

### Environmental predictor variables

We downloaded environmental variables (Table 2) including slope, aspect, elevation, and solar radiation with 30-second (ca. 1 km) spatial resolution from WorldClim version 2 datasets [72]. We processed the variables using the ‘extract by mass’ tool in ArcGIS (version 2.4) to produce environmental layers. We selected climatic variables as predictors, taking into consideration that *Agave* productivity, physiological performance, and cultivation can be reliably predicted using temperature, precipitation, and solar radiation [73, 74].

**Table 2:** Environmental variables derived from monthly temperature and rainfall values. The variables were selected from the WorldClim 2 database to generate dryland-farming MaxEnt models. All the environmental variables analyzed for the study can be found in (S4 Fig).

Environmental predictor variables:
Bio 2 = mean diurnal range (mean of monthly (maximum temperature—minimum temperature))
Bio 7 = temperature annual range (maximum temperature—minimum temperature)
Bio 8 = mean temperature of wettest quarter
Bio 15 = precipitation seasonality (coefficient of variation)
Bio 16 = precipitation of wettest quarter
Bio 19 = precipitation of coldest quarter
Aspect
Elevation
Slope

After variables were processed, we analyzed multicollinearity using cross-correlations (Pearson correlation coefficient *r*) using ENMTools in RStudio (S4 Fig). To ensure minimal correlation between the most relevant environmental factors, also identified as predictor variables for distribution-suitability modeling, we used values <0.7 in the Pearson correlation analysis as suggested by [75, 76]. Nine continuous environmental variables, which were included in our analysis, are summarized in Table 2. The MaxEnt analysis determines the level of importance, or the test gain, of each variable. The test gain is particularly important in analyzing the variables to explain model trends.

### Model performance and validation

We used the cross-validation method [77] to assess the model performance of the two dryland-farming systems. We analyzed the models with the ‘leave-one-out’ method, also known as jackknife analysis [50]. The ‘leave-one-out’ method is a systematic approach that tests the individual contribution of each data point to the overall model performance. The method has been used in ecology and conservation biology when dealing with small size data sets, which have ≤ 25 samples. This method has been tested widely in different ecological-niche models to build species-distribution models of large areas with limited data occurrences [50, 59, 78].

In addition, we used the area-under-the-curve (AUC) approach to validate the modeled dryland-farming and wild *Agave* population distributions, which were generated using MaxEnt. The AUC method estimates the ratio between true-positive rate and the false-positive rate, which allows for predicting and ranking locations using only presence data [47, 50]. We used a binomial test to evaluate the prediction-potential accuracy of MaxEnt [48, 50, 54].

We evaluated the importance of environmental-predictor variables in the individual models. MaxEnt evaluates distribution models by tracking which environmental variables most influence the predicted distribution in the model [46]. The MaxEnt algorithm uses the gain function to estimate the model predictivity likelihood. The gain function estimates the ratio between occurrences and predicted sites in the environmental variables selected to create the model [46, 47]. We examined which variables contributed most to building prediction-distribution models of dryland-farming systems and for various *Agave* species. We then built response curves of the environmental predictor variables (Fig 4), which provided the most significant contributions to the model.

### Comparisons of dryland-farming models with *Agave* species

In order to understand the relationship between the extent of dryland-farming systems and *Agave* species in the region, we paired models using the identity test in ENMTools [71], also called permutation-analysis assessment, to statistically test if the predicted model distributions of dryland-farming systems and *Agave* species overlapped in the region. MaxEnt creates probability-habitat-suitability scores as functions of the environment across the landscape [79]. Likelihood of species similarities can be quantified in the distribution models by analyzing suitability scores of each individual pixel (e.g., spatial gain) in the MaxEnt output [46]. We evaluated the estimated levels of closeness or habitat occupancy overlapping between species distribution in both dryland-farming systems and wild *Agave* species using the permuted *I*-statistic (permutation test) or identity test (Table 3), which is widely used in understanding ecological-niche models [80, 81]. The *I*-statistic uses the occurrence data to randomly extract subsamples from MaxEnt maps and then reanalyzes the distribution to parameterize species and to calculate overlapping. This method allows researchers to quantify how similar or different the distributions are between two species, and how they are related with respect to their habitat predictability [80]. We considered significant differences between species-habitat distributions (non-identical niches) when the observed (non-permuted) *I*-statistic values were below the critical (permuted) threshold (5%) in ENMTools [71, 80, 81].

**Table 3:** Suitability and overlapping statistical assessment using permutation tests.

Suitable areas comparisons	Observed *I*	5% critical *I*
Rock piles vs. grassland	0.52	0.47
Rock piles vs. *Agave palmeri*	**0.39**	**0.46**
Rock piles vs. *Agave parryi*	**0.21**	**0.34**
*Agave palmeri* vs. *Agave parryi*	**0.42**	**0.92**
*Agave palmeri* vs. *Agave angustifolia*	**0.34**	**0.64**
*Agave parryi* vs. *Agave angustifolia*	**0.17**	**0.71**
Grassland vs. *Agave angustifolia*	0.45	0.43

Summary of the observed *I* values and critical *I* values calculated using the permutation tests. Significant differences between predicted suitable areas occur when observed *I* values are lower than the 5% critical *I* values, indicating non-identical suitable areas for dryland-farming and *Agave* species. Bold values indicate significant differences between paired suitable areas calculated in the permutation test.

Permutation analysis assesses the similarities of the predicted areas using MaxEnt modeling [48, 50]. A permutation test or identity test is a comparative analysis of two probability distributions from two MaxEnt models to statistically evaluate how close these models are in their predicted geographic range [71]. Significant differences between models can be inferred when the observed value is lower than the critical value [80, 81]. While there are limited examples of using this kind of analysis for characterizing suitability models in agriculture [45, 82, 83], this analysis can be used to understand the differences and similarities between the environments and to assess their dryland-farming suitability.

#### Software

The *Agave* dryland-farming and *Agave* population distribution models were created in the Java program MaxEnt 3.4.4 [48]. We used Dismo package [84] and ENMTools to analyze model overlap, species occurrences, and species-distribution maps. We analyzed preprocessing of species occurrences and climatic layers using ENMeval [70] and ENMTools in RStudio Version 3.6.3.

## Results

### Model performance and validation

**Fig 2:**
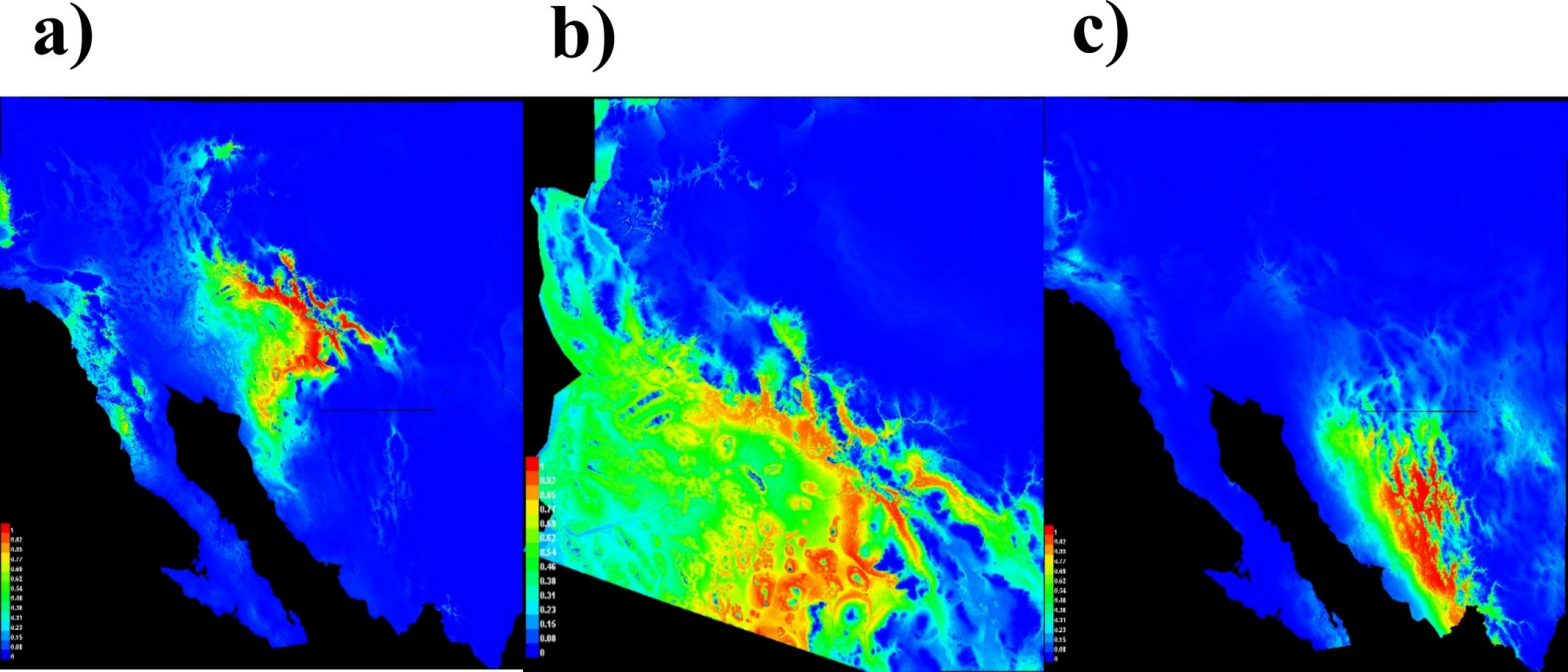
Suitability models for rock piles in the Sonoran Desert region (a), Arizona, USA (b), and for *Agave* grasslands in Sonora, Mexico (c). Coloration in red indicates high suitability potential for rock piles in (a) and (b). Coloration in red in (c) indicates high suitability for grasslands.

**Fig 3:**
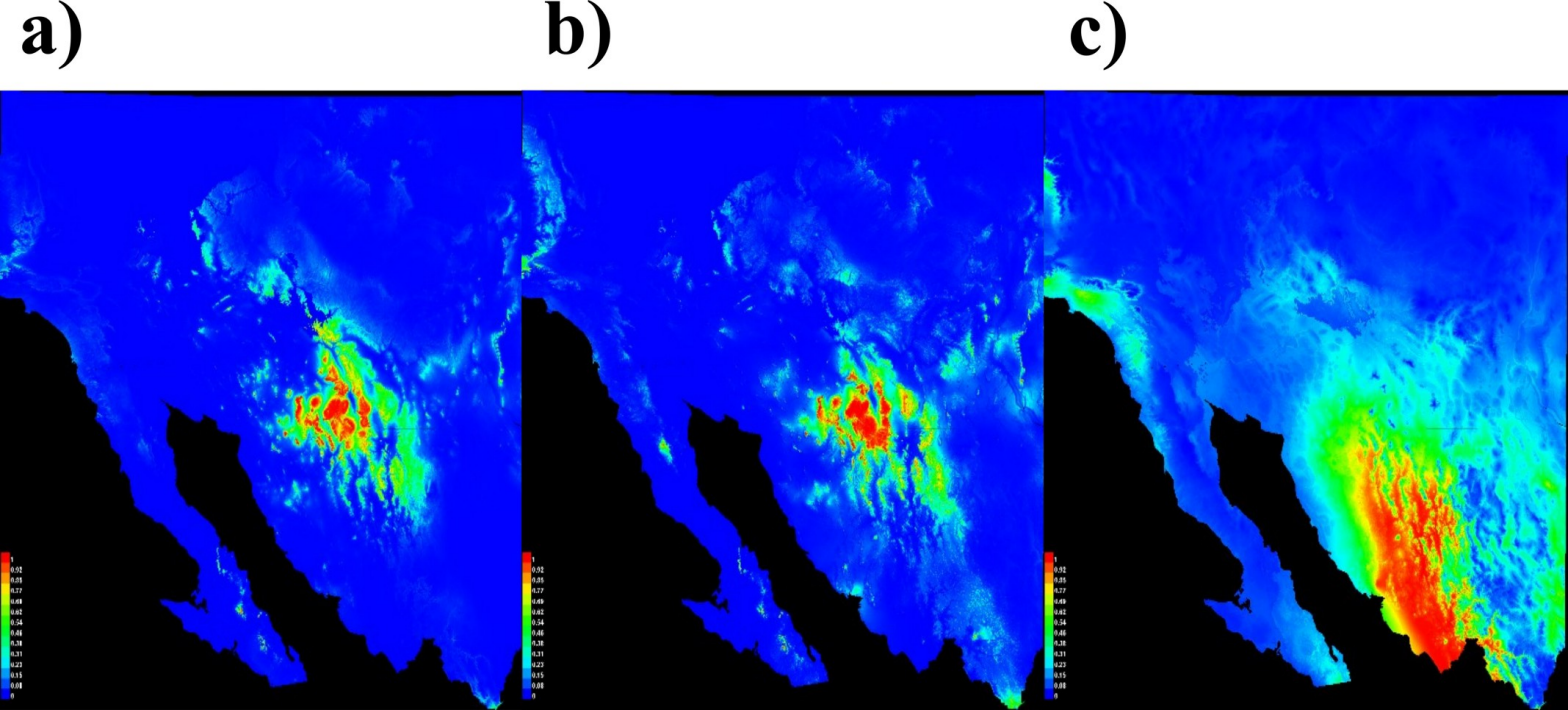
Suitability models of *Agave palmeri* (a), *Agave parryi* (b), and *Agave angustifolia* (c) in the borderlands of Arizona, USA and Sonora, Mexico. Red color indicates high suitability for *Agave* species.

The models for the two dryland-farming systems (rock piles and *Agave* grasslands) and also for wild *Agave* species yielded AUC values between 0.96 and 0.99 (Table 4), suggesting that MaxEnt performed well in constructing prediction models for rock piles and grasslands suitable for *Agave* cultivation across Arizona and Sonora [85]. The AUC is a model performance measurement and validation tool generated by the MaxEnt algorithm [46, 51, 52, 54]. Relatively high AUC model values (≥0.90), suggests that the distributional extent of *Agave* dryland-farming can be forecasted, even with limited samples [59]. Ardestani *et al*. [52] suggested that AUC values from MaxEnt models with values greater than 0.90 are optimal, with values ranging between 0.80–0.90 and 0.70–0.80 considered to be moderate and acceptable. Area-under-the-curve values of dryland farming and *Agave* species were estimated to be <0.95 (Table 4). These values illustrate the power of MaxEnt to create models of the potential extent of rock piles and grasslands for *Agave* cultivation in the borderlands of Arizona and Sonora [85] (Figs 2 and 3).

**Table 4:** Summary of permutation importance, via MaxEnt analysis, for each individual variable in the dryland-farming and *Agave*-species models.

	Environmental permutation importance (%)								
Species	Slope	Aspect	Elevation	Bio 2	Bio7	Bio 8	Bio 15	Bio 16	Bio 19
Rock piles	1	0	64.6	0	0	0.2	19.7	14.3	0.2
Grassland	0	0.3	10	0	4.8	3.1	72.6	0.1	9.1
*Agave angustifolia*	1.2	0.1	7.2	0	0	7.7	0	83.8	0
*Agave palmeri*	0.4	0	8.1	12.7	0	43.6	16.3	5	13.9
*Agave parryi*	1.5	0	30.7	8.1	0	15.5	31.5	1	11.6

Overall, the jackknife analysis indicated differences between variable importance for rock piles and *Agave* grasslands and between *Agave* species (Fig 1 and Table 4). For rock piles in Arizona, the elevation range was the variable that contributed the most (64.6%) to build the distribution of dryland farming across the state and in the borderlands with Sonora. Suitability models for *Agave* grasslands in Sonora, indicated that the variable, precipitation seasonality, contributed the most (72.6%) to construct the model. *Agave parryi* had a similar association with precipitation and elevation as observed for rock piles, indicating precipitation seasonality and elevation as the two most influential environmental variables to the suitability model of this *Agave* species in the region (Table 1). Precipitation in the wettest quarter of the year (i.e., June, July, and August) was the main variable in predicting potential suitable areas for *A*. *angustifolia* in Sonora (Fig 3A and 3B). In contrast, the model of *A*. *palmeri* indicated that mean temperature in the wettest quarter was the main variable to predict suitable areas for this *Agave* species in the borderlands of Arizona and Sonora.

We assessed the accuracy of MaxEnt in creating suitability models for dryland farming and *Agave* species using a binomial-probability test (Table 4) [46, 50]. This nonparametric analysis has been suggested when using small sample sizes to test whether predictability of a suitability model is proportional to the samples used to create the model [50]. The predictability analysis for rock piles using the binomial-probability test was marginally significant, yielding a *p*-value of 0.08, indicating that there were a sufficient number of rock-pile fields included in the analysis to construct a reliable rock-pile model to determine the potential extent of this dryland-farming system in Arizona and adjacent areas in Sonora (Fig 2A). Similarly, the predictability of the model for *Agave* grasslands yielded a statistically significant *p*-value of 0.03, showing that MaxEnt created a reliable model of the potential distributional extent of dryland farming for cultivating *A*. *angustifolia* in Sonora. The predictability of the models for *A*. *parryi* and *A*. *palmeri* yielded *p*-values of > 0.1, which were not statistically significant, thus indicating low predictability of MaxEnt for these two species. Overall, however, model results indicate fairly accurate predictability to identify areas for rock piles and *Agave* grasslands in the region (Fig 2A–2C).

### Suitable areas for dryland-farming and *Agave* cultivation

The rock-pile model suggested potential suitable areas for rock piles in central and southern Arizona, primarily in the southern borderlands of Arizona, including Pima County and the reservation lands of the Tohono O’odham (Fig 2A and 2B). In central Arizona, potential suitable areas span through Gila, Pinal, and Yavapai Counties. The rock-pile model yielded potential locations from south-central Arizona to northwestern Sonora. The *Agave*-grassland model (Fig 2C) indicated that the regions of central and southern Sonora have the highest potential to cultivate *Agave*, particularly *A*. *angustifolia*.

The locations with the highest suitability for *A*. *palmeri* and *A*. *parryi* in the Sonoran Desert are concentrated in the borderlands between Arizona and Sonora (Fig 3A and 3B). However, the suitability of both species decreases towards southern Sonora. The model suggests that suitable areas to cultivate *A*. *angustifolia* in southern Sonora center in the Fuerte-Mayo region, while the northern mountain range of the Sierra Madre Occidental in Sonora in the northern boundary of the designation-of-origin region for bacanora is less suitable (Fig 3C) (S2 Fig).

### Importance of environmental variables to predict suitable areas

Our results suggest that none of the individual variables showed similar test-gain values when compared with the full-model test-gain values (Table 4). This indicates that all variables are necessary to predict suitable areas for *Agave* species and potential dryland-farming areas in the region.

**Fig 4:**
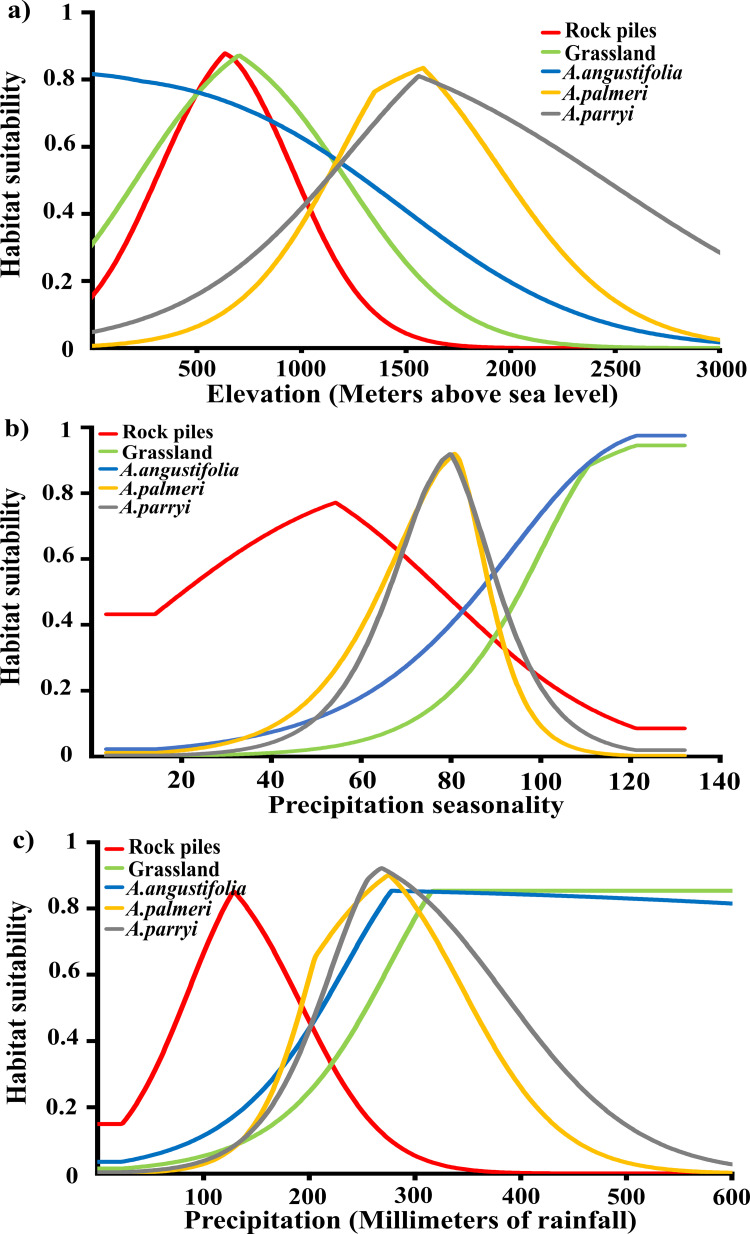
Habitat suitability for rock piles, grasslands, and *Agave* species (*A*. *angustifolia*, *A*. *palmeri*, and *A*. *parryi)* during the wettest season in a given year in terms of three environmental variables: (a) elevation, (b) precipitation seasonality, and (c) annual precipitation.

The most suitable areas to employ rock piles had elevations between 650–1200 m above sea level (Fig 4A). These areas also had precipitation seasonality values ranging between 40–70% (Fig 4B). Precipitation seasonality is an estimation of the deviation of the monthly variation of annual rainfall, which is also called coefficient of variation of annual precipitation, and is an index of inter-annual rainfall variability. The index describes fluctuations and likelihood of precipitation in a region. It is calculated as the ratio of the standard deviation of the monthly total precipitation to the mean monthly total precipitation expressed in percentage. The higher the percentage of this index, the higher variability of rainfall in a region. Estimated rainfall for suitable rock-pile areas was estimated at 100 mm with no more than 150 mm in the wettest season of the year, which is during the North American monsoon season (i.e., June, July, August) (Fig 4C). In addition, the most suitable areas to employ grasslands had elevations between 700–1300 m above sea level (Fig 4A) and precipitation around 300 mm during the summer months (Fig 4C), which accounts for more than 80% of the deviation of the monthly variation of the precipitation over the course of a year (Fig 4B).

The most suitable areas for wild *Agave* species had elevations between 200–1000 m above sea level for *A*. *angustifolia* and between 1400–1500 m above sea level for *Agave palmeri* and *Agave parryi* (Fig 4A). In addition, rainfall in suitable areas for *A*. *angustifolia* was estimated in areas with 80% of annual mean precipitation above 300 mm of rainfall. Suitable areas for *A*. *palmeri* are those that received around 200 mm of rainfall during the monsoon season. Suitable areas for *A*. *parryi* areas can be found where precipitation averages 250 mm of rain during the monsoon season (Fig 4B and 4C).

### Comparisons between dryland-farming systems and areas suitable for cultivation of *Agave*

#### Predicted distribution of rock piles and grasslands cultivated with *A*. *angustifolia*

The permutation test to compare distributions of rock piles with grasslands cultivated with *A*. *angustifolia* yielded 0.52 for the observed value and 0.47 for the critical value (Table 4). Slight differences between observed and critical values of rock-pile fields and grasslands models using the permutation test suggest that although distributions of the two dryland-farming systems were predicted in different areas (Fig 2), the differences between their predicted geographic ranges were indistinguishable using the permutation test.

#### Rock piles paired with *Agave* species

Rock piles paired with *A*. *palmeri* and *A*. *parryi* in the permutation test indicated significant differences in their geographic distributions (Table 5). Analysis of the proximity of predicted distributions of the rock-pile model with *A*. *palmeri*, using the permutation test, yielded an observed value of 0.39 and a critical value of 0.46. Distribution of rock piles compared with *A*. *parryi* distribution yielded an observed value of 0.21 and a critical value of 0.35. These results suggest that the predicted areas in the suitability models (Figs 2 and 3) occur in different areas within the borderlands of Arizona and Sonora.

**Table 5:** Summary of niche models of individual species for dryland-farming and *Agave* species with test gains of all the variables used to build the distribution models with only one variable.

Dryland- farming and *Agave* species	Model test gain		Model accuracy	Test gain for individual variables								
	Test AUC	Full model	*p-*values	Slope	Aspect	Elevation	Bio 2	Bio7	Bio 8	Bio 15	Bio 16	Bio 19
Rock piles	0.99	3.14	0.08	0.15	0.63	0.15	0.59	0.62	0.43	0.43	0.17	0.28
Grassland	0.98	2.85	0.03	0.60	0.61	0.21	0.47	0.61	1.94E-01	0.29	0.56	0.31
*Agave angustifolia*	0.96	1.88	0.1	0.56	0.62	0.33	0.54	0.65	0.29	0.39	0.58	0.64
*Agave palmeri*	0.96	2.59	0.9	0.73	0.58	0.19	0.46	0.18	0.12	0.22	0.12	0.20
*Agave parryi*	0.98	3.41	0.3	0.97	0.70	0.32	0.63	0.38	1.82E-01	022	0.26	0.28

The importance and contribution of the variables to the full model can be extracted from the estimated gain of each of them of each variable relative to the full model. Abbreviated environmental variables are defined as follows: Bio 2 = mean diurnal range (mean of monthly (maximum temperature—minimum temperature)), Bio 7 = temperature annual range (maximum temperature–minimum temperature), Bio 8 = mean temperature of wettest quarter, Bio 15 = precipitation seasonality, Bio 16 = precipitation of wettest quarter, Bio 19 = precipitation of coldest quarter, and Bio 19 = precipitation of coldest quarter.

#### Comparisons of Agave angustifolia with A. palmeri and A. parryi

Comparisons of *A*. *angustifolia* with *A*. *palmeri* and *A*. *angustifolia* with *A*. *parryi* indicate that the observed values were lower than the critical-value permutation scores (Table 5), suggesting that predicted suitable areas for *A*. *palmeri* and *A*. *parryi* species occur in areas different from those with *A*. *angustifolia*.

#### Cattle-grazed grasslands paired with *A*. *angustifolia*

Suitability models for grasslands and for *A*. *angustifolia* (Figs 2C and 3C) indicate that wild *A*. *angustifolia* overlaps with predicted grasslands areas. The analysis of *A*. *angustifolia* with *Agave* grasslands, which yielded an observed value of 0.46 and a critical value of 0.44, did not show differences in their distributions, suggesting that they occur in similar areas in Sonora. In southern Sonora, we found that there was considerable overlap between grassland areas and predicted suitable areas of wild *A*. *angustifolia* (Fig 3C).

## Discussion

### Predictability of MaxEnt dryland-farming models

We found that MaxEnt is a powerful tool for modeling suitable areas for *Agave* dryland farming, particularly using rock piles, as was found for determining suitability of Ak-Chin dryland features [53]. MaxEnt models provided a first look of the potential geographic extent of suitable area for dryland farming using rock piles in the borderlands of Sonora and Arizona. Likewise, using current climate data, MaxEnt created models for *A*. *angustifolia* cultivation in cattle-grazed areas across Sonora. These models highlighted potential areas for *Agave* cultivation along the edges of the designation-of-origin region for mescal bacanora [21]. In addition, MaxEnt models highlighted suitable areas in the borderlands of Sonora and Arizona of two *Agave* species (*A*. *parryi* and *A*. *palmeri*) with potential to be used in the *Agave* agricultural industry [56]. *Agave* dryland-farming models, which incorporate data related to rock piles and cattle-grazed fields, provide an overview of the potential areas to cultivate *Agave* in lands with limited access to irrigation water.

Although rock piles are a pre-Columbian technology not currently used in the borderlands, they can be used to cultivate *Agave* and harvest rainfall water in the current climate. More recently, mescal farmers in Puebla, Mexico, where rain is scarce, harvest *Agave marmorata* in soils with high superficial stone content [86]. Farmers in the region have observed that *Agave* grown in rocks increases mescal palatability. In regions with 200 mm or less of annual rainfall, such as in semi-arid areas of Guanajuato, Mexico and South Africa, mescal farmers successfully cultivate *Agave salmiana* and *Agave americana* [87, 88]. Since *Agave* dryland-farming practices generally do not use tillage, these techniques can enhance soil properties by increasing storage of rainwater [89]. Our study also highlighted the potential of diversifying use of the land by using dryland farming and endemic *Agave* species in the *Agave* agricultural industry. Dryland farming and use of endemic *Agave* species, such as *A*. *palmeri* and *A*. *parryi*, can provide options that complement the bacanora industry in Sonora [42].

Amongst environmental and climate factors, temperature and moisture act as major variables interacting together for modeling suitable regions for *Agave* dryland farming [32]. Garcia-Moya *et al*. [74] suggested the importance of identifying optimal regions with environmental and climate conditions to support sustainable *Agave* cultivation in current and future climate change. Forecasting dryland farming is key for *Agave* cultivation in the borderlands between the U.S and Mexico due the current significant increase in temperature and drought intensity, which is expected to continue in upcoming decades [43, 44]. Our models highlight regions within the borderlands of suitable climates for dryland farming, particularly those with elevations where temperature and rainfall likely increase productivity of *Agave* [30, 73, 74] (Fig 4).

#### Distribution and suitable areas for *Agave* dryland farming

Our models predicted the distribution of rock piles throughout the Sonoran Desert region and grasslands across the Sierra Madre in Sonora (Fig 2A and 2B). Rock piles were predicted in the Sonoran Desert mainland, predominantly in southern and central Arizona (Fig 2B). The predicted distribution for rock piles included areas in the southeastern portion of Arizona in the borderlands with northwest Sonora, particularly near the municipalities of Caborca and Altar (Fig 2A). *Agave*-grassland farming was predicted throughout most of the state of Sonora, suggesting that *Agave* cultivated in grasslands are likely to succeed in more diverse environments and climates outside of the Sonoran Desert [3, 15, 30].

Our models highlighted two particular regions within Sonora with suitable climates to cultivate *A*. *angustifolia* using dryland farming: 1) the original designation-of-origin region for mescal bacanora [90, 91]; and 2) the Fuerte-Mayo region. The original designation-of-origin region for mescal bacanora [15, 90] includes 35 municipalities in the Sierra Madre (S2 Fig). However, in southern Sonora, the region of Fuerte-Mayo, which includes the municipalities of Huatabampo, Navojoa and Alamos, is currently outside of the boundaries of the designation-of-origin region for mescal bacanora (Figs 2C and 3C). The Fuerte-Mayo region was originally described by Gentry in 1972 [3] to be where there are several taxa within the *A*. *angustifolia* species complex. Given that mescal bacanora is produced traditionally and crafted in the mountain ranges in the Sierra Madre Occidental in Sonora, this region was not considered part of the designation-of-origin region for bacanora [90, 92]. Also, *A*. *angustifolia* is not commercially cultivated in the region [15, 93]. The Mexican designations of origin differ from those used in Europe, in that in small geographic regions and traditional products are named after towns, such as for mescal bacanora, which likely was named after the town of Bacanora in northern Sonora [94, 95]. However, conflict could arise if the designation-of-origin region for bacanora is expanded, potentially creating rivalries within the *Agave* agricultural industry in Sonora [18, 96]. Given the diversity of *Agave* species in Sonora, the mescal bacanora industry can be complemented with other traditional spirit drinks produced artisanally across Sonora [3, 55]. Besides bacanora, different mescals have historically been produced in Sonora along the edges of the designation-of-origin region for bacanora [18].

#### Suitable environments and climate for rock piles vs. *Agave* grasslands

Our models indicated environmental differences in predicted distributions of rock piles and *Agave*-grassland areas within the region (Fig 2A and 2C). Apparent lower precipitation in predicted suitable areas for rock piles relative to predicted suitable areas for grasslands were extracted from the models (Fig 4B and 4C). For south-central Arizona and the southeastern portion of the borderlands with Sonora, precipitation is lower than along the Sierra Madre and southern Sonora where *Agave* grasslands were predicted. Rock piles and grasslands also differed in elevation (Fig 4A). Although predicted rock piles and grassland areas had similar elevations between 500–1000 m above sea level, suitability for rock piles decreased at 1500 m above sea level (Fig 4A). Our models indicated that grasslands occupy a range of elevations in Sonora between 500–2000 m in the Sierra Madre Mountains [15, 30]. However, areas for rock piles are more likely to be found in mesic environments of the Sonoran Desert in south-central Arizona, which are generally found in lower elevations, which is consistent with rock-pile fields in central Arizona located between 600–900 m [4].

Hill slope and air temperature were factored into past models of productivity of agaves in the region [32, 74, 97]. Nobel and Hartsock [98] reported that productivity of *Agave deserti* was correlated with slope-face direction. Cervantes-Mendívil *et al*. [15] observed in grazing areas, which were cultivated with *A*. *angustifolia*, that plants adapted better and survived more often in sites with low temperatures above 1°C than in sites with temperatures between -2 to -8°C in the Sierra Madre in Sonora. Nuñez-Noriega *et al*. [30] suggested that in cattle-grazing areas, temperatures at elevations between 800–1200 m are ideal for *A*. *angustifolia* cultivation. Fish and Fish [2, 4] suggested that in rock-pile fields in mesic environments with elevations between 600–900 m, *Agave* species are less susceptible to low temperatures. Our models suggest that while slope and temperature are important environmental factors for site selection for cultivation and for plant survival, they carry less influence than precipitation and elevation in predicting the potential distributional extent of dryland-farming within the region (Table 4 and Fig 4).

While previous models assessed suitable cultivation areas for *Agave* using a theoretical multi-criteria approach in the region (e.g., climate and productivity indexes and GIS) [32], such models did not include dryland farming as a factor to forecast *Agave*-cultivation areas. However, the extent of dryland farming predicted in our models indicated similar areas for *Agave* cultivation in Arizona as the models reported by [32]. These results suggest southern and central Arizona are regions with high potential for dryland farming. Additionally, our models indicated suitable environments and climate with high potential for application of *Agave* dryland farming, particularly using rock piles in the borderlands of Arizona and Sonora (Fig 4).

### Differences between dryland-farming-system and *Agave*-species models

For *Agave*-grassland farming in Sonora, permutation analysis indicated overlap with the native range of *A*. *angustifolia*, suggesting that the environments for cultivation in grasslands are similar to environments where *A*. *angustifolia* naturally occurs. Conversely, the analysis of our models showed little or no correlation between suitable environments for rock piles and *A*. *palmeri* and *A*. *parryi*, which occur in the borderland region of Arizona and Sonora (Table 5). Comparisons of rock piles with these two *Agave* species suggest that their predicted ranges likely occur outside of the predicted areas for rock piles. In addition, the predicted suitable climate for these two species is likely found outside existing rock-pile fields in south-central Arizona.

Two *Agave* species, *Agave murpheyi* and *Agave sanpedroensis*, have been reported to have been cultivated by the Hohokam using rock piles [4, 8, 35]. Similarly, *A*. *marmorata*, which is harvested from the wild in Puebla, Mexico, has been observed to grow well in areas with high rock content [86]. Evidence of historic uses of *A*. *palmeri* and *A*. *parryi* has been found at archaeological sites (S3 Fig) [33, 64]. However, these two *Agave* species have been found outside the native distributions of *Agave* species that were cultivated using Hohokam rock piles [8].

Based on the analysis of suitability models for rock-pile fields and agaves (Fig 4), our results indicate that *A*. *palmeri* and *A*. *parryi* require more moisture than where rock-pile fields are generally located [99]. Likewise, our results (Fig 4B) suggest that these two species occur in more moist environments than Hohokam rock-pile fields sites in south-central Arizona region [27]. Wild species of *A*. *palmeri* and *A*. *parryi* have been reported in similar environments within the borderlands of Arizona and Sonora [8, 27, 33]. In addition, these two endemic species are conventionally used to produce mescal in the borderlands [66]. Research on applications of dryland farming to cultivate these two species is needed. Similarly, more research is needed to characterize historical cultivation of these two *Agave* species (S3 Fig) [33]. Knowledge about the suitability and similarities of dryland farming and *Agave* species, based on their climatic requirements and distributions, can be used as a tool to identify more *Agave* species that potentially could be cultivated using dryland farming in the future. Although using permutation tests allowed us to analyze overlapping between dryland farming areas and the native ranges of *Agave* species, accuracy of this method might be affected by sample sizes [59], which requires further experimentation.

### Comparison of *Agave angustifolia* to *Agave palmeri* and *Agave parryi*

*Agave angustifolia* is the most abundant *Agave* species in Sonora [3, 15, 30]. Forecasting the suitable distribution of *A*. *angustifolia* and the possible overlap with the native ranges of other *Agave* species, such as *A*. *palmeri* and *A*. *parryi*, provides a clearer sense of geographic boundaries between these species within the region and with dryland farming in the borderlands of Arizona and Sonora. Upper distributional limits of *A*. *angustifolia* were predicted in the north-central part of Sonora, adjacent to where *A*. *palmeri* and *A*. *parryi* naturally occur (Fig 3A–3C).

#### Comparisons of *Agave palmeri* vs. *Agave parryi* models

Despite the fact that the predicted suitable models showed similarities between wild *A*. *palmeri* and *A*. *parryi* species (Fig 3A and 3B), the permutation test indicated that distributions of these two species are different in their predicted environments within the region. Our results comparing suitable distribution models for these two *Agave* species using the permutation test showed that when extracting permutation scores from these models using ENMTools, the observed value (0.42) was lower than the critical value (0.94), indicating differences in their predicted distributions. Field work is needed to estimate the extent of the distributional range of these two *Agave* species. Field observations by Nabhan *et al*. [33] suggests that some wild *A*. *parryi* species, such as the subspecies *A*. *parryi* var. *huachucensis*, overlap with wild *A*. *palmeri* species.

#### Future research to forecast dryland-farming and *Agave* cultivation

The economic potential of *Agave* as a drought-tolerant crop for arid regions has been extensively characterized [10–12, 26, 74]. However, despite the substantial research on the impacts of climate change on *Agave*, little research has been conducted on *Agave* dryland-farming cultivation in the borderlands of Arizona and Sonora [6]. Current patterns of climate changes pose challenges to the *Agave* agricultural industry in Sonora. In order to continue producing mescal bacanora, research is needed to find sustainable methods to produce *A*. *angustifolia* [30, 42]. Finding endemic *Agave* species that complement the current *Agave* industry in Sonora is also crucial. By diversifying *Agave* cultivation in Sonora and the borderlands with Arizona, producers can expand beyond production of mescal to develop other products derived from *Agave*, such as feedstock, syrups, biofuel, synthetic drugs, fructans, and saponins [10, 26, 100]. An increase in the cultivation of *Agave* in the borders could also boost the economy of the region [10]. The *Agave* industry in Sonora is gaining a stronger economic foothold in the EU and U.S. markets [21, 26]. Moreover, agronomical research for exploring *Agave* as crop in the borderlands needs a collaborative effort between Mexico and the U.S. in the context of current climate change [30].

This is the first time MaxEnt modeling has been used to predict potential areas suitable for *Agave* dryland farming in Arizona and Sonora. MaxEnt has primarily been used in ecological-niche studies to find species-specific locations [46]. The purpose of our study was to use MaxEnt to identify areas for potential *Agave* cultivation. A similar approach was reported by Healy *et al*. [53] to assess ancient dryland farming in New Mexico. Although MaxEnt can estimate suitability-distribution models with limited samples [59], we found our results to be constrained because the exact geographic boundaries of our models cannot be confirmed without extensive field work. More specifically, two factors likely affected our results: 1) rock-pile fields were less abundant in the region relative to the abundance of grassland fields, and 2) limited information is available regarding the two dryland-farming systems in the borderlands. Only a small number of rock-pile fields and *A*. *angustifolia*-grasslands fields have been agronomically studied in Arizona and Sonora [15, 29, 30]. Depending on sample size and geographic extent selected to produce suitability models, two kinds of models can be predicted using MaxEnt: widespread models (i.e., large sample size) and narrow-range models (i.e., minimal to medium sample size) [59]. Based on the limited availability of samples for this study, our models fit in the category of narrow-range models. As such, interpretation of our results should be treated with caution. Future studies likely need a more refined search of dryland farming in the region to better address the ratio of predicted and known samples in the borderlands region. In addition, experiments with reciprocal common gardens with rock piles cultivated with agaves would enhance our understanding of the potential application of rock piles in *Agave* production.

## Conclusions

The ecological-niche-modeling software, MaxEnt, identified potential areas for *Agave* cultivation in the borderlands of Arizona with Sonora. Although there are constraints in our study, MaxEnt prediction maps and environmental variable analyses of *Agave* dryland farming and *Agave* species models provide a strong foundation for future research on *Agave* cultivation in the region.

The use of rock piles, which have similar properties to mulch to retain moisture and buffer soils from high temperatures, is a promising water-conserving method to cultivate *Agave* in the Sonoran Desert. Our research determined potential areas for *Agave* rock-pile cultivation beyond the known archaeological range in the Tucson-Phoenix Basin in the southwestern portion of Arizona in the borderlands with Sonora. Potential areas with suitable climates for *Agave* cultivated using grasslands were mainly found outside the Sonoran Desert region, but throughout other parts of the state of Sonora, including two regions: 1) the designation-of-origin region for bacanora (S1 Fig) and 2) the Fuerte-Mayo region (Figs 2C and 3C). Our models identified southern Sonora, primarily the Fuerte-Mayo region, as a potential area in the outer limits of the range of the designation-of-origin region to cultivate *A*. *angustifolia* for bacanora production, particularly in grasslands [15]. Additionally, our models highlighted southern Arizona and the borderlands of north-central Sonora as a suitable region for cultivating *A*. *palmeri* and *A*. *parryi* (Fig 2A and 2B). Our models suggest that *A*. *angustifolia* might be found in the same areas identified for potential dryland farming of agaves in grasslands (Fig 3A and 3B). Based on the predicted distribution models of rock piles and grasslands, our models suggest that dryland farming has potential to be implemented in Sonora and Arizona to cultivate agaves. Future agricultural and agroecological studies on rock piles and *Agave* grasslands are necessary to understand their application in these areas.

## Supporting information

S1 Fig Map of the borderland states of the U.S. and Mexico. The area highlighted in grey was the study area used to create suitability models for *Agave* dryland farming and *Agave* species. The shapefiles and data used to construct the map were obtained from GADM [https://gadm.org/index.html].
(TIF)

S2 Fig Designation of origin for mescal bacanora in Sonora, Mexico. The shapefiles and data used to construct the map were obtained from GADM [https://gadm.org/index.html].
(TIF)

S3 Fig *Agave parryi* plant growing in an ancient rock pile at an archaeological site in Casas Grandes, Chihuahua, Mexico. Published under a CC BY license, with permission from Michael T. Searcy, original copyright 2022.
(TIF)

S4 Fig Analysis of collinearity of environmental variables from the WorldClim 2 database to generate dryland-farming MaxEnt models. Highlights in red indicate the variables selected for the study.
(TIF)
