# Correction: Ecological-niche modeling reveals current opportunities for *Agave* dryland farming in Sonora, Mexico and Arizona, USA

**DOI:** 10.1371/journal.pone.0343010

**Published:** 2026-02-13

**Authors:** Hector G. Ortiz Cano, Robert Hadfield, Teresa Gomez, Kevin Hultine, Ricardo Mata Gonzalez, Steven L. Petersen, Neil C. Hansen, Michael T. Searcy, Jason Stetler, Teodoro Cervantes Mendívil, David Burchfield, Pilman Park, J. Ryan Stewart

There are errors in the author affiliations. The correct affiliations are as follows:

Hector G. Ortiz Cano^1^, Robert Hadfield^2^, Teresa Gomez^2^, Kevin Hultine^3^, Ricardo Mata Gonzalez^4^, Steven L. Petersen^2^, Neil C. Hansen^2^, Michael T. Searcy^5^, Jason Stetler^2^, Teodoro Cervantes Mendívil^6^, David Burchfield^2^, Pilman Park^7^, J. Ryan Stewart^2^

**1** The Holden Arboretum, Kirtland, Ohio, USA, **2** Department of Plant and Wildlife Sciences, Brigham Young University, Provo, Utah, USA, **3** Department of Research, Conservation and Collections, Desert Botanical Garden, Phoenix, Arizona, USA, **4** Department of Animal and Rangeland Sciences, Oregon State University, Corvallis, Oregon, USA, **5** Department of Anthropology, Brigham Young University, Provo, Utah, USA, **6** Instituto Nacional de Investigaciones Forestales, Agrícolas y Pecuarias (INIFAP), Campo Experimental Costa de Hermosillo, Sonora, México, **7** Floriculture Research Division, National Institute of Horticulture and Herbal Sciences, Rural Development Administration, Jeonju, South Korea
