# Supplementary figures and images for: Ecological-niche modeling reveals current opportunities for Agave dryland farming in Sonora, Mexico and Arizona, USA
Source: PLoS One. 2023 Jan 20;18(1):e0279877. doi: 10.1371/journal.pone.0279877 (PMC9858763; doi:10.1371/journal.pone.0279877)

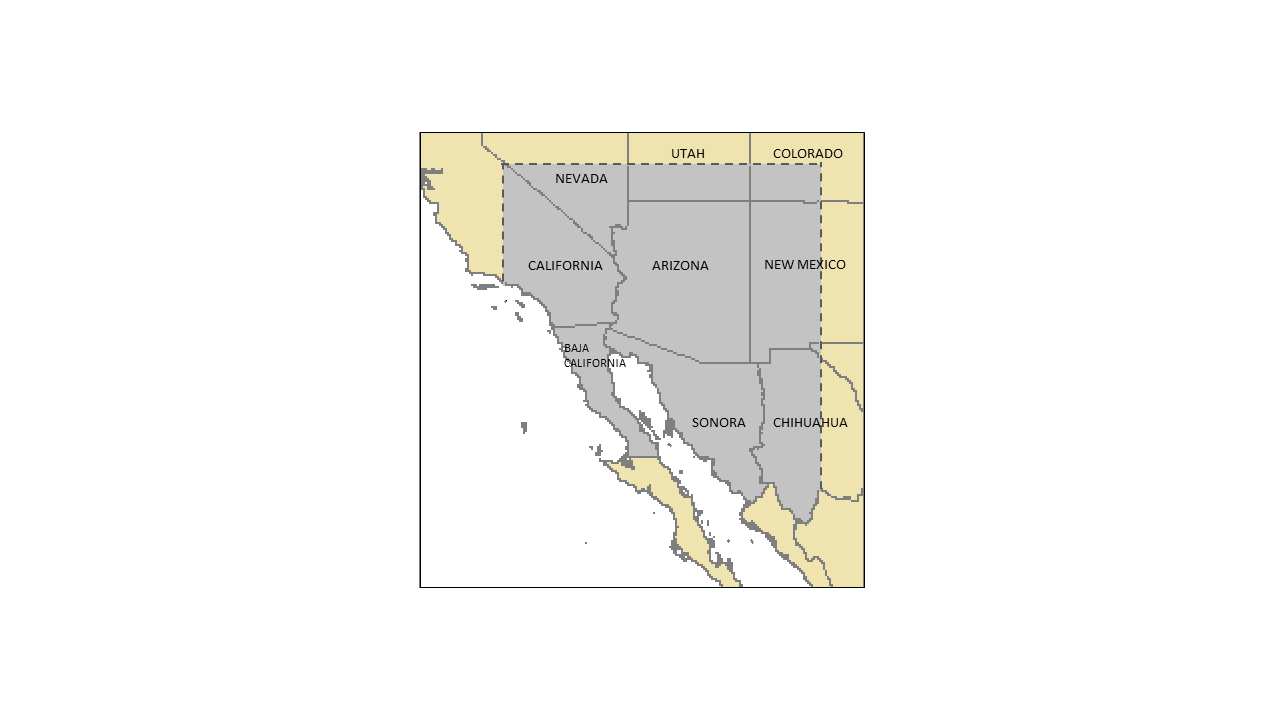

Supplement: S1 Fig — The area highlighted in grey was the study area used to create suitability models for Agave dryland farming and Agave species. The shapefiles and data used to construct the map were obtained from GADM [https://gadm.org/index.html]. (TIF) [file pone.0279877.s001.tif]

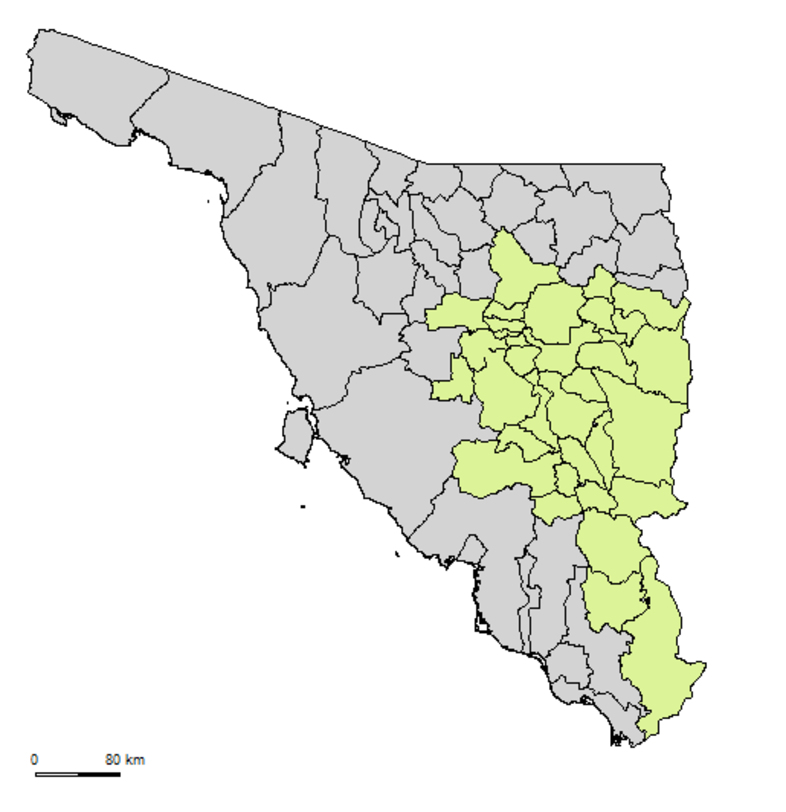

Supplement: S2 Fig — The shapefiles and data used to construct the map were obtained from GADM [https://gadm.org/index.html]. (TIF) [file pone.0279877.s002.tif]

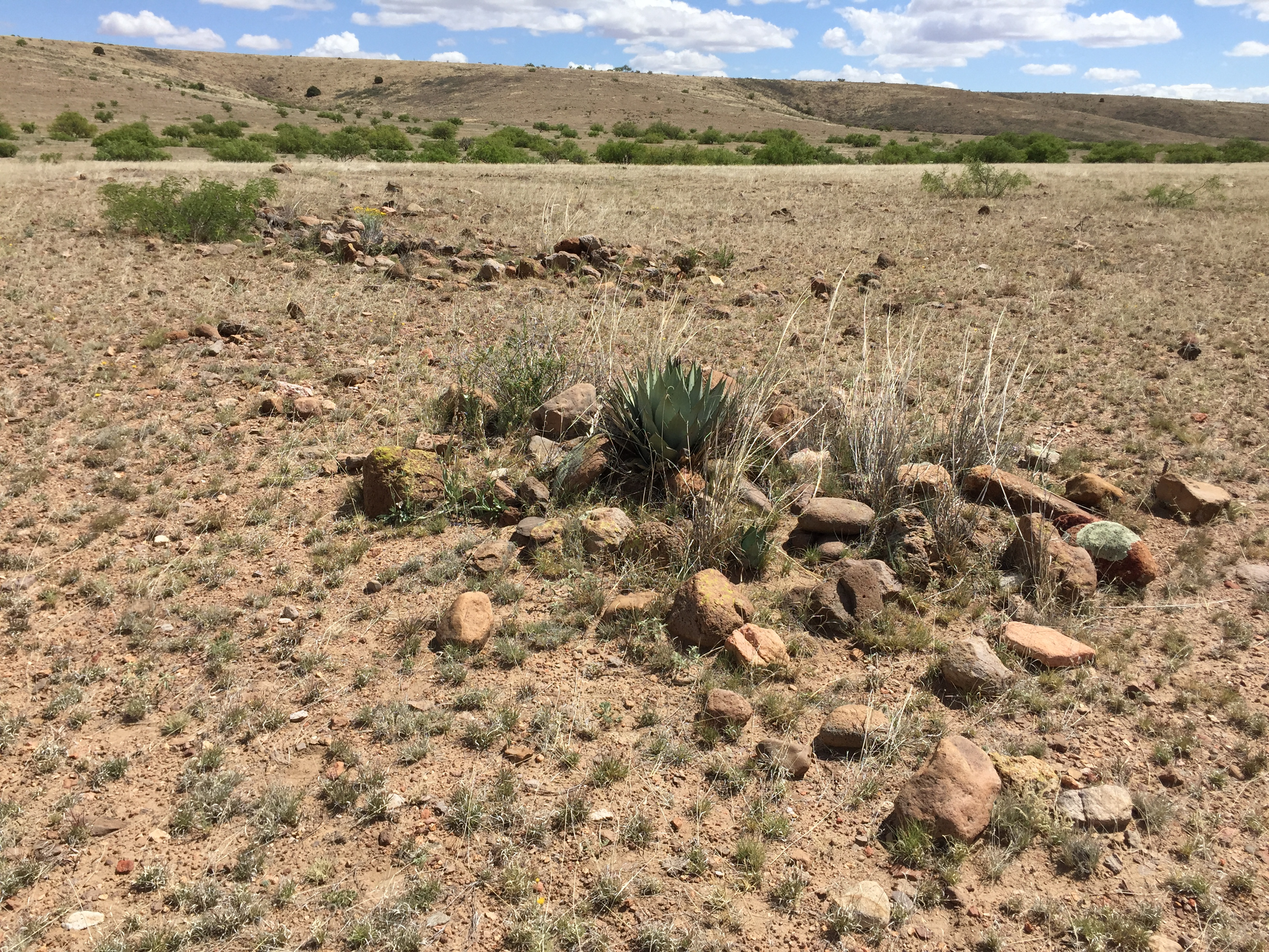

Supplement: S3 Fig — Published under a CC BY license, with permission from Michael T. Searcy, original copyright 2022. (TIF) [file pone.0279877.s003.tif]

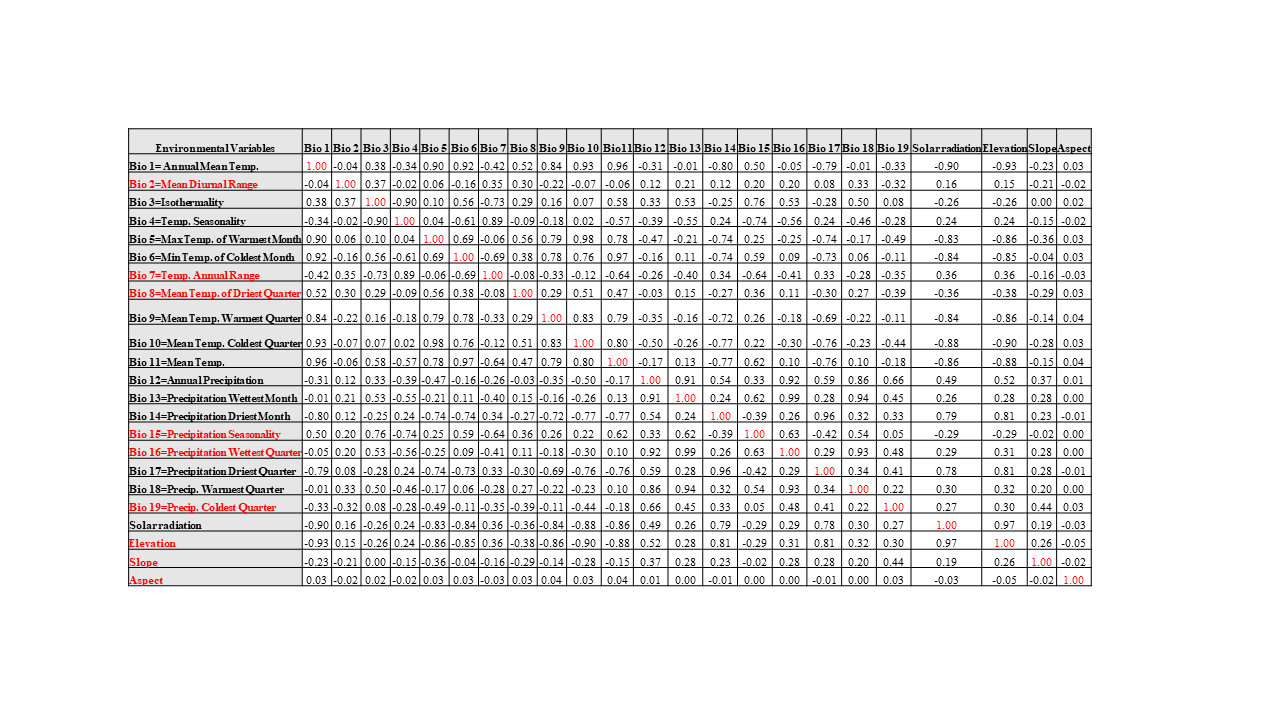

Supplement: S4 Fig — Highlights in red indicate the variables selected for the study. (TIF) [file pone.0279877.s004.tif]
